# Navigating foster care: how parental drug use and caregiver attitudes shape children’s mentalization processes—an exploratory longitudinal follow-up study: study protocol

**DOI:** 10.3389/fpsyg.2024.1295809

**Published:** 2024-06-13

**Authors:** Nadja Springer, Brigitte Lueger-Schuster

**Affiliations:** ^1^Dialog – Individuelle Suchthilfe, Vienna, Austria; ^2^Unit of Psychotraumatology, Faculty of Psychology, University of Vienna, Vienna, Austria

**Keywords:** mentalization-based treatment, reflective functioning, foster care, substance use disorder, parenting

## Abstract

**Background:**

The current research concept of mentalization is used in the study to clearly identify affective and cognitive abilities of the caregiver-child dyad with the aim of compensating deficits on both sides with psychological-psychotherapeutic strategies.

**Methods:**

The objective of this explorative, longitudinal intervention study is to provide an in-depth understanding of the psycho-social background of 30 children aged 6–12 years living in institutional or family-centered foster care. Data will be collected at three time points: before, after and 12 months after participating in the newly developed group intervention, which intends to address the particular needs of children of drug abusing parents living in foster care in the latency period. The study is conducted at the Faculty of Psychology of the University of Vienna in collaboration with the Association “Dialogue” (Verein Dialog). The treatment duration spans 5 months, during which two specifically trained psychotherapists conduct 10 group sessions for children and three group sessions for foster caregivers. All statistical analyses will consider the type of data available. Therefore, the primary outcome of the study will be assessed via the Friedman test due to the ordinal dependent variable as it is the non-parametric alternative to the one-way ANOVA for repeated measures. In addition, the Mann–Whitney U test is used to compare differences between two independent groups (children living in institutional foster care vs. family foster care). To assess potential correlations regarding the child and caregivers’ capacity to mentalize, Spearman correlations (ρ) are conducted. To examine the secondary outcome, apart from the methods previously outlined, we will also utilize qualitative thematic analysis.

**Discussion:**

The present study uses the current research concept of mentalization to identify affective and cognitive abilities of the caregiver-child dyad with the aim of compensating deficits on both sides with psychological-psychotherapeutic strategies. There are some limitations of the study to mention: the small sample size does not allow to generalize the results. Due to the lack of a comparison group, a randomized control study (RCT) was not conducted. The authors are aware of these limitations. However, the studies’ findings, will help to deduce research questions for further studies.

## 1 Introduction

Foster care is government-subsidized and -regulated temporary care for children who have been removed from their families for reasons of abuse and neglect. Children can be placed either in family or residential care. While family foster care includes arrangements of children living with unrelated foster parents (nonrelative foster care), with relatives (kinship care), or with families who plan to adopt them (foster/adopt homes), residential programs encompass a type of living in out-of-home care placement in which specialized services for youth with emotional and behavioral problems or other special needs are provided in a highly structured environment. It was estimated that in 142 countries about 2.7 million children aged 0–17 could be living in institutionalized care worldwide (Petrowski et al., 2017).

In Austria, in 2021 a total of 12,871 minors were living in foster care (of which 31.5% only in Vienna): 61.3% of the children and adolescents were placed in institutional group care, while 38.7% were accommodated with foster families (Österreich, 2022). In terms of age, 44.3% of children in foster care, were between the ages of 6 and 14. Children placed into institutional care usually live together with 8–10 other children, cared for by a team of social pedagogues, each of them having one main contact person, called “caregiver.”

Although estimates vary widely, a detailed literature search suggests that parental substance use disorder plays a major role in the child welfare system (Seay, 2015).

Maltreatment, such as neglect, and placement into foster care are considered as traumatic affecting children’s immediate and future psychosocial development and mental health (Cicchetti and Toth, 1995; Bowlby, 1998; Cicchetti et al., 2006). Children placed in foster care might have experienced distressing feelings such as confusion, anxiety and sadness, due to the unfamiliar or previously experienced situation to which they are adapting as a result of their placement (Bruskas, 2008). High rates of internalizing problems (e.g., anxiety, depression), externalizing problems (e.g., aggression, impulsivity), poorer social skills, and lower adaptive functioning were also reported among foster children compared to children who did not experience replacement (Webb et al., 2010; Jones Harden et al., 2014).

Specifically, those children who experienced trauma frequently revealed underdeveloped mentalizing capacities (Ostler et al., 2010). Muller et al. (2012) specified that their ability to cope with physical, or emotional traumas highly correlated with the perceived quality of their current relationships, and that traumatic experiences had a major impact on attachment behaviors of out-of-home children toward their foster caregivers. As a result of the children’s adverse experiences with parental care, they may be inclined to avoid forming new and supportive relationships. This creates a complex situation for all individuals involved, including both the foster caregiver and the children. On the other side, foster parents and institutional caregivers know that foster care is mostly not permanent. This issue may interfere with attachment formation to the foster parents/caregivers (Åkerman et al., 2020).

The ability to mentalize plays a crucial role in individuals’ coping abilities with traumatic events (Pecora et al., 2005; Szilagyi et al., 2015). Mentalization can be understood as “the ability to understand the actions by both other people and oneself in terms of thoughts, feelings, wishes and desires” (Bateman and Fonagy, 2016, p. 3). By involving the internal regulation of emotions through thoughts, children as well as adults who are able to mentalize are more resilient and able to tolerate feelings of anger, fear, shame, and distress resulting from adversity and trauma (Allen et al., 2008; Brockmann and Kirsch, 2010; Ostler et al., 2010; Ensink et al., 2017; Giusti et al., 2021).

Research revealed a strong association between insecure attachment representations and struggles of drug addicted parents in responding to their children’s emotional cues (Grienenberger et al., 2005; Slade et al., 2005), which in turn interfere with the ability to form secure attachments and mentalizing skills in children (Madigan et al., 2007; Bammens et al., 2015). However, most studies have focused mainly on the characteristics of the child, neglecting the parents’ variables (Schmidt and Schimmelmann, 2015).

Pediatricians reported that children of drug-using families were often distressed by their parents’ substance use. They often had to take on parental roles, due to their parents’ incapacity. As they usually have blamed themselves for their parents’ behavior and felt responsible for their wellbeing (Smith and Wilson, 2016), they tended to keep the situation secret and not show their distress in public (Morrison et al., 1996; Hoffmann and Su, 1998). They also tended to self-stigmatize and live in fear of “failing” in the same way as their parents did (Matthews et al., 2017). Within addiction care, children of drug abusing parents are not always seen as individuals in need due to the fact, that the focus stays on the consuming adult. Nevertheless, detecting problems in children early as well as prompt implementation of prevention or treatment programs, would increase the likelihood of the child growing up safely (Van de Meer et al., 2019). Moreover, children of parents with Substance Use Disorder (SUD) experience a notably higher incidence of physical, emotional, and sexual abuse, as well as emotional or physical neglect (McGlade et al., 2009; Altshuler and Cleverly-Thomas, 2011). Therefore, foster care children of drug abusing families, place an additional strain on the healthcare system, thus more research identifying factors enabling children and adolescents to enhance their mental wellbeing and healthy development, as well as cost-effective treatment options, is needed.

Parental attitudes represent the primary social influence encountered by the child during their formative years (Zunich, 1966). As Farmakopoulou and Baltsioti (2024, p. 319–320) emphasize clearly: “Foster carers play a crucial role in this institution, and their experiences, perceptions, and relationships are vital for the wellbeing and development of the children under their care. Understanding foster carers’ views on factors that support or hinder successful fostering is essential for grasping the dynamics of foster care.”

Children’s ability to mentalize can be best observed during the latency period in child development. The latency period takes place between the age of 6 and 12 and is characterized by a number of new developmental challenges, such as finding a place in a peer group, realizing cognitive activities, and compliance to the rules of the family and the community. Individuals’ ability to cope with these challenges largely depends on the ego-function development, reflected, e.g., in emotional control, ability to deal with frustration and being able to reflect on the other’s perspective (Akhtar, 2009; Dejko et al., 2016).

Studies reported that a parent’s ability of reflective functioning (RF), as mentalization is operationalized, predicted children’s attachment security and mentalization ability (Grienenberger et al., 2005; Slade et al., 2005; Allen et al., 2008), and various aspects of a child’s short- and long-term development (Fonagy et al., 1991; Slade, 2002; Steele and Steele, 2008). Improvements in RF related to mothers with SUD were associated with improvements in caregiving quality (Åkerman et al., 2020). The studies mentioned above looked in samples of biological or foster parents. The majority of interventions that have been developed to support foster carers has been criticized for their lack of focus on improving the capacity of carers to respond to the child’s related needs while it is obvious that keeping the needs of a child in mind is the best support for every child’s development RF and social skills, such as reflexibility and social orientation, help every carer to understand behavior as a respond to emotional needs instead of just seeing “naughty” or “bad” behavior and the child itself feels understood and valued (Adkins et al., 2018; Midgley et al., 2019).

RF is defined as the ability to mentalize in the context of close, interpersonal relationships. With the words of Fonagy et al. (1998), RF helps us “to distinguish inner from outer reality, pretend from “real” modes of functioning as well as intra-personal mental and emotional processes from interpersonal communications” (Fonagy et al., 1998, p. 4). A recent study, showed that a group of brief psychoeducational parenting intervention increased RF in foster parents and improved parental sensitivity while decreasing children s’ internalizing behavior (Adkins et al., 2022).

Research on attachment and mentalization in substance-abusing families is emerging. While studies exist on mentalization in substance abusing mothers (e.g., Suchman et al., 2006; Pajulo et al., 2012; Suchman, 2016), research on mentalization or reflective functioning in children of substance-abusing parents is still lacking, and evidence suggest the specific need for interventions aimed to address experiences and feelings associated with foster care in children (Ostler et al., 2010).

Mentalization-based treatment (MBT) programs to increase ego-functions have been developed and implemented for multiple indications such as borderline personality disorders, drug addiction, and for children with parents suffering from mental health disorders (e.g., Bateman and Fonagy, 2008, Bruskas, 2008; Suchman et al., 2010). They aimed to understand and improve the individual’s ability to reflect upon their own and others’ feelings, thoughts, and desires. When applied to children and their caregivers, MBT focus on replacing destructive habitual ways of feeling and acting by improving mentalizing abilities, together with the individual’s emotional wellbeing, and interpersonal skills (Mayes, 2012).

A Narrative Systematic Review (Midgley et al., 2021) examined the range of mentalization-based interventions for children in middle childhood (6–12 years). According to this review, a relevant number of mentalization-based interventions have been conducted in the context of fostering and adoption, but the childrens’ mentalizing capacity was hardly ever assessed as an outcome and children from drug abusing families are still lacking as target group. This lack was already mentioned in previous publications (Jacobson et al., 2015; Midgley et al., 2017), but did not change so far.

## 2 Methods/design

### 2.1 Aims

The first aim of this study is to explore the development longitudinally of the children’s mentalization ability at three measure time points: before, after and 12 months after attending a newly developed group MBT-intervention. It is an adapted version of Midgley et al. (2017). Mentalization-Based Treatment for Children (MBT-C), which addresses the particular needs of children of substance using parents, who live in foster care during middle childhood, integrating the psycho-social background of children living in foster care. Second, we will explore the mentalization capacity of the caregivers, their attitudes on drugs and drug addiction, and their social skills (e.g., social orientation and reflexibility) with regard to the impact on the children’s mentalization at the beginning of the intervention to better understand whether it influence the development of mentalization over time.

### 2.2 Participants

In the study 30 children living in foster care (institutional or foster home) and their actual main foster caregiver will be included (*N* = 60).

Children’s inclusion criteria for participation are: (1) age between 6 and 12 years, (2) stable mental health status (e.g., no psychotic state), (3) living in foster care for at least 6 months. A minimum of time in a stable and persistent placement needs to be considered to assure the children’s ability to reorganize their internal world and behavioral outcomes (Cassibba et al., 2023). Caregiver’s inclusion criteria are (1) child lives in the same household and (2) 18 years of age or older. Children are excluded from the study if presenting neurological, cognitive, and/or psychiatric problems, and/or difficulties reported by their caregiver.

Children in middle childhood, aged between 6 and 12, are very common as primary target of mentalization-based treatments. However, middle childhood has received only limited attention regarding the ability to mentalize (Midgley et al., 2021).

### 2.3 Measures

Relevant biographical data are collected at baseline within a clinical interview. They included the children’s age at the first out-of-home placement, substance use dependency syndrome of biological parents and the number of caregivers since out-of-home placement. Data Research Topic is conducted by specifically trained clinical psychologists and psychotherapists.

#### 2.3.1 Primary outcome

Caregiver and child Reflective Functioning (RF) are assessed by a licensed coder using the Reflective Functioning Scale (RFS) (Fonagy et al., 1998; Shmueli-Goetz et al., 2011) to interpret predefined sections of the Adult Attachment Interview (AAI) (George et al., 1985), and using the Child Reflective Functioning Scale (CRFS) (Target et al., 2001) to interpret the Child Attachment Interview (CAI) (Shmueli-Goetz et al., 2008) transcripts. The AAI and the CAI, semi- structured clinical interviews focus on the subject’s attachment experiences with their parents during childhood. Some questions in the AAI (e.g., “Why did your parents behave as they did during your childhood?,” “Do you think your childhood experiences have an influence on who you are today?”) and some questions in the CAI (e.g., “Do your parents sometimes argue? How do they feel? Why do you think they do that?” or “What happens when your mum gets cross with you or tells you off? How do you feel?”) require reflective functioning (RF), while others allow it. According to Fonagy et al. (1998), RF occurs when the interviewee shows (1) awareness of the nature of mental states, (2) an explicit effort to tease out the mental states underlying one’s own and others’ behavior and (3) the tendency to recognize developmental aspects of mental states over time or (4) mental states in relation to the interviewer. After rating each identified passage of the AAI, an overall score is assigned to each interview ranging from −1 (negative RF) to 9 (exceptional RF) (Taubner et al., 2013). The same procedure will be conducted for the CAI.

After rating each identified passage of the CAI, an overall classification is assigned to the interview, using a hierarchical approach, and distinguishing between 10 levels of RF, ranging from −1 (negative RF) to 9 (exceptional RF) (Ensink et al., 2013).

Validation studies of the RFS (Fonagy et al., 1998; Ensink et al., 2015) proved discriminant and predictive validity and good interrater reliability. Temporal stability of children’s RF was shown to be high over a 3-month period and adequate over 12 months (Ensink, 2004).

#### 2.3.2 Secondary outcomes

To investigate the children’s mental health, their clear-thinking ability, and their dynamic personality structure, as well as the caregiver’s attitudes on drugs and drug addiction and their social skills (e.g., social orientation and reflexibility) the following measures are chosen:

- The Child Behavior Checklist (CBCL/6-18R) is a parent report form to screen for emotional, behavioral, and social problems in children, based on the DSM-IV (Esser and Hänsch-Oelgart, 2018). A DSM-5 oriented version is not yet available. For the aim of the present study the presence of the following psychiatric disorders from the Diagnostic and Statistical Manual of Mental Disorders IV (American Psychiatric Association, 1994) were assessed: anxiety, oppositional defiant disorder, conduct problems, somatic problems, affective problems, and attention deficit disorder. The profiles show raw scale scores derived by the sum of each item. The scales are quantitatively scored in terms of gender- and age-specific t-scores with cut points for normal (t-scores from 50 to 64), borderline (t-scores from 65 to 69), and clinical (t-scores from 70 to 100) ranges (Döpfner et al., 2014). The CBCL is widely used in clinical research and diagnostical practice and shows good results regarding reliability and validity (Nakamura et al., 2009).
- Raven’s Colored Progressive Matrices (RCPM) measures clear-thinking ability and is designed for young children aged 5–10 years and older adolescents. The RCPM is internationally recognized as a culture-fair test of nonverbal (fluid) intelligence. The test consists of 36 items clustered in 3 sets (A, Ab, B), with 12 items per set. Most items are presented on a colored background to make the test visually stimulating for participants. The RCPM produces a single raw score that can be converted to a percentile based on normative data, designating the following categories: Performance Level 1 “well above average” (>95 percentile); Performance Level 2 “above average”(percentiles 75–95); Performance Level 3 “average” (percentiles 25–75); Performance Level 4 “well below average” (percentiles 5–25); Performance Level 5 “intellectual disability” (percentiles <5) (Bulheller and Häcker, 2002). In daily clinical practice, RCPM test is used as one of the best general intelligence measures (Smirni, 2020).
- The social orientation and Reflexibility subscales of the 33 items- Inventory of Social Competences (ISK; Inventar sozialer Kompetenzen) by Kanning (2009) is used to assess the caregiver’s social skills. The raw-data analysis is standardized by means of an evaluation form. The 17 raw scores of the primary scales and the four total raw scores of the secondary scales are transformed into stanine scores with the help of standard tables (Kanning, 2009). The reliability of the instrument can be rated as satisfactory to good. Numerous results from several individual studies on convergent and discriminant validity are reported (Scherp, 2010). For this study, we focus on the two subscales social orientation and reflexibility.
- The Patte-Noire (“black paw”) -Test is a French thematic-projective test for children from 6 to 12 years: a storytelling test through the character of the little pig Patte Noire, used in children’s clinical assessment, to elicit themes related to the child’s perceptions of the relationships between parents and children, and siblings and to obtain unconscious material. The test is administered according to Corman (1972) criteria in two main parts: telling a story and identifications. The child is asked to look at 17 different panels that show pigs in social situations, to choose one or more of them, to tell a story about “Patte Noire” and also to tell with which character on the panels he/she identifies—if possible. While working on the story, the child has to put him/herself in the situation described by the panels. In each panel one little pig is marked with a black paw. Every panel stimulates one of the following themes: orality, anality, oedipal issues, aggression, dependency-independence, guilt, black spot, inverted sexes, nurturing father, ideal mother. For the purposes of this study, 7 sheets out of the total 17 comprising Corman’s Patte Noire Test have been selected to take a look at the identifications in terms of attachment relations as it was already conducted and published by Ballus et al. (2019). The 7 selected pictures express clearly attachment relations in terms of attachment theory (Corman, 1977; Ainsworth et al., 1978; Bowlby, 1982; Yarnoz, 1993) and were selected in order to elicit information about the children’s attachment experiences, for example panel N°4: “the Cart.” Patte Noire (PN) dreams that a farmer is taking some piglets away in a cart. PN’s parents and two other piglets watch the scene. Theme of separation and loss. The childrens’ identifications on panel N°4 (“the cart”) in relation to the foster care situation has our special interest looking at the children’s identifications with the little pig (“Patte Noire”) or with “No One” or with somebody outside the scenery on the panel. This test is highly recommended by child psychoanalysts and is frequently used for clinical psychological diagnosis in addition to clinical psychological tests.
- Attitudes to Drug Use (ADU) (Harmon, 1993). A German version, translated and retranslated by expert native speakers, of a non-standardized 12-items paper-pencil-questionnaire is used to assess the caregiver’s attitudes to drug use on a 5-point-rating scale ranging from 1 (strongly disagree) to 5 (strongly agree). The questions are formulated as statements such as “using illegal drugs can be pleasant” or “trying drugs is losing control of your life.” Computation of Indices: Items (a), (d), (e), (i), (k), were scored “5” for “strongly agree” to “1” for “strongly disagree,” while the other items (−) were scored in the opposite way (1 for “strongly agree” to “5” for “strongly disagree”). To obtain an attitude score for each individual, the scores for each item were summed and divided by the number of items answered. A score of 5.00 indicates a completely positive attitude toward drugs while a score of 1.00 indicates a completely negative attitude. In accordance with Hulin et al. (2001), the internal reliability of this scale is within a very good range (α = 0.89).

### 2.4 Procedure

The study is conducted by the Faculty of Psychology of the University of Vienna in collaboration with the NGO Dialog.^1^ The study was approved by the Ethical Committee of the University of Vienna (#00295). All procedures performed in the study follow the ethical standards of the institutional and/or national research committee and are in agreement with the Helsinki Declaration and its later amendments or comparable ethical standards. Written informed consent is obtained from the foster parents or legal guardians, and assent from children is gained as well.

Officially registered with the DRKS (German Clinical Trials Register) #DRKS00027868, the trial is displayed on the public website. It has been submitted to the WHO and is searchable through its meta-register.^2^ Caregiver and child Reflective Functioning (RF) are assessed by a licensed coder using the Reflective Functioning Scale (RFS) (Fonagy et al., 1998; Shmueli-Goetz et al., 2011) to interpret predefined sections of the Adult Attachment Interview (AAI) (George et al., 1985), and using the Child Reflective Functioning Scale (CRFS) (Target et al., 2001) to interpret the Child Attachment Interview (CAI) (Shmueli-Goetz et al., 2008) transcripts.

The trial site is the outpatient center of the NGO Dialog in Vienna (A), where the mentalization-based group intervention was developed and takes place. This NGO specializes in prevention and treatment of substance use disorders for more than 40 years.

**Figure 1:**
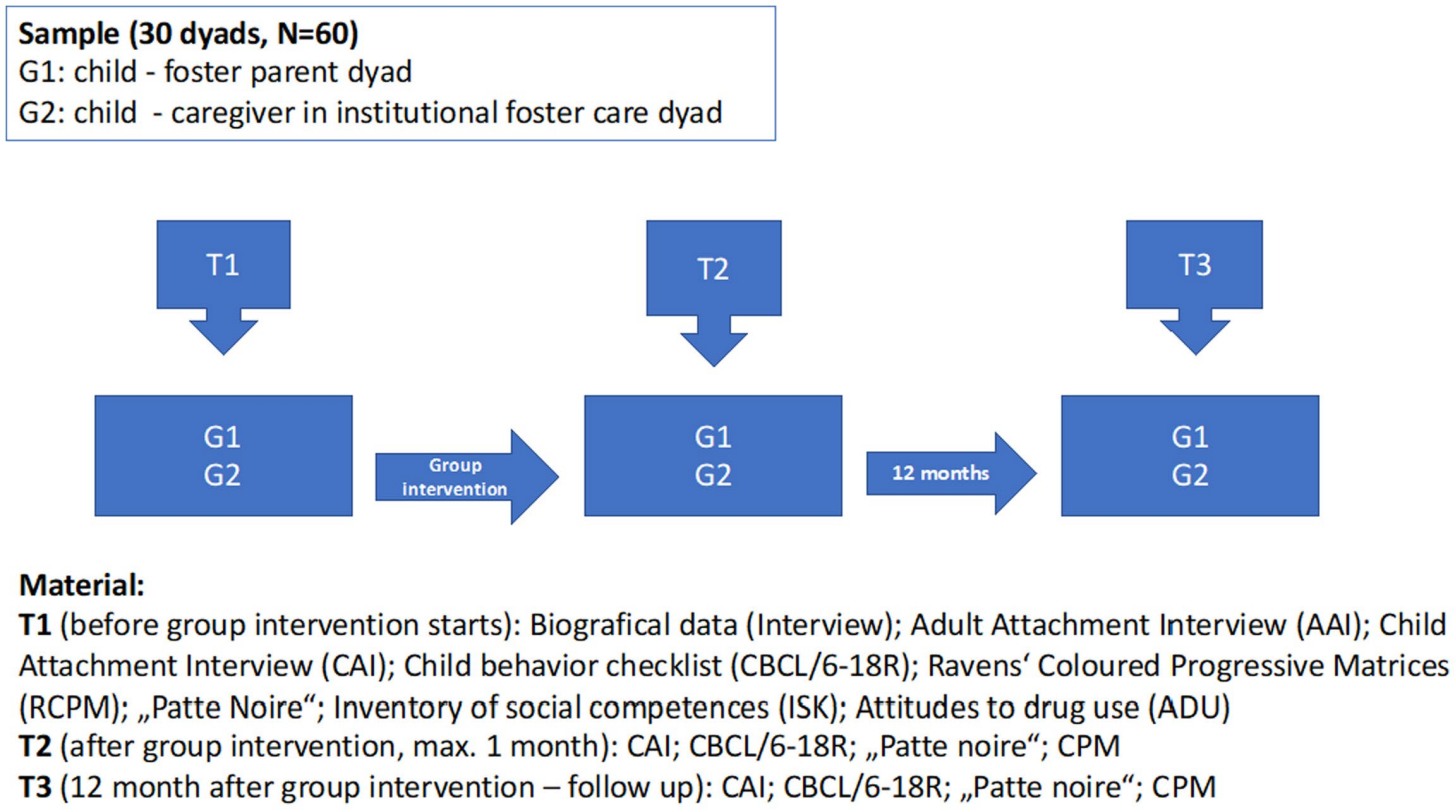
Study flow chart. Sequence of steps from T1 to T3.

Participants follow a 20-week mentalization-based group intervention aiming at increasing knowledge about drug addiction and at enhancing social and affective skills in children (6–12 years) of drug abusing families living in foster care. The participation in the study is voluntary. Data are collected at baseline (T1), within 1 month after treatment termination (T2) and at 12 months follow-up (T3) (Figure 1).

### 2.5 Mentalization-based group intervention

Findings from a cross-European study conducted in 2007 on Domestic Violence and Abuse among Adolescents from Alcohol-affected Families indicated that adolescents expressed the value of being in relation with others who have undergone similar experiences or have faced similar challenges within their families. They perceived these interactions beneficial specifically in realizing that they were not alone in these circumstances (Velleman et al., 2008). The mentalization-centered group model offers a secure and contained environment, akin to a *relational laboratory*, in which children can safely investigate their thoughts and emotions regarding their real-life situations and emotional challenges (Malberg, 2012).

The Mentalized-Based Group Intervention (MBGI) was adapted from the MBT-C by Midgley et al. (2017) to the particular needs of children of drug abusing parents living in foster care in middle childhood. In accordance with other short-term interventions, such as “SMART” (Fearon et al., 2006) or “Trampolin” (Wiedow et al., 2011), ten group sessions for children and three group sessions for the foster caregivers are provided by specifically trained psychotherapists in a period of 5 months (school semester). The facilitators have attended trainings at the Anna Freud Centre in London (UK) on *“Mentalizing and Mentalization Based Treatments with Children, Young People and Families (MBT CYP)”* as well as *“Reflective Parenting (MBT-RP).”* Each session lasts about 90 min. The first encounter with the caregivers takes place after the first children’s group session to get to know each other, to obtain a first impression of the group by the psychotherapists, and to introduce mentalization processes to the caregivers in order to make them part of the children’s support. The second caregiver session is, scheduled halfway through the intervention. It is used to reflect on the ongoing treatment and its impact on the children’s daily lives. The final group session with the caregivers serves as a review of the experience and a preview of future intervention opportunities. The three caregiver sessions include information on parental licit/illicit drug abuse background focusing on the caregiver-child-interaction, along the process described by Fonagy and Allison (2014): (1) development of a therapeutic context in which the children/caregivers feel understood to reduce the sense of epistemic trust; (2) reemergence of the participants’ capacity to mentalize, as they “find their mind in the mind of the therapist”; (3) being aware of and interested in the content of the psychoeducational work of the therapists and to benefit from the group as a place to share experiences.

For the children, the 10 group sessions follow a ritualized procedure: there are predefined contents, such as drug addiction in general, the children’s situation in foster care, their feelings toward their biological parents and their foster caregivers. Since group dynamics in the sessions are never predictable, there is always time for “open topics,” brought up by the children. Group sessions are always conducted in the same room, which does not contain too many distracting elements but is large enough to allow for physical games like throwing and catching a ball or play “Emotion-Charade,” where children try to nonverbally pantomime a pre-selected emotion to each other. The material provided to the children involved painting, drawing and sketching tools. Children are invited to talk or to express themselves and their feelings by using available materials. Drawing is particularly useful, feels to be less intrusive and threatening than the request to talk about one’s experiences (Gardner and Harper, 1997).

The aims of the group sessions are (1) restoring the frame to think and talk about highly affective feelings such as anger, fear and shame, (2) building up a small and safe mentalizing community to explore mental states and giving sense to the behavior of oneself and others, (3) discussing the individual meaning and consequences of the experience of parental substance use or parents being “high” on licit/illicit drugs, and (4) providing new ego-strengthening skills.

The ritualized procedure contains a welcome circle that allows space for current events or feelings, planned psychoeducational inputs, and a special feedback method to recap what was discussed in each session and gather the children’s opinions on it.

The psychoeducational input always contains an interactive play or method.

As outcome of this Mentalization-Based Group Intervention, we expect a decrease in children’s psychiatric symptoms as there is evidence from previous studies on the effects of Mentalization-Based Interventions (Åkerman et al., 2020; Dalgaard et al., 2023). Furthermore, we expect an increase in the RF Score (capacity to mentalize). It has been shown that the RF Score can change as a result of psychotherapy, especially in psychodynamic oriented psychotherapy. These results have been observed, using the AAI to gain the RF Score (Katznelson, 2014).

### 2.6 Statistical plan and data analysis

Sociodemographic data will be used to characterize the sample and to provide an in-depth look into the participant’s psycho-social background. Frequencies and means are derived for demographic variables.

Thresholds for the measures will be used to describe each case, and to provide descriptive statistics, such as frequencies, mean and standard variation (SPSS 27.0), for each group: caregivers, children living in foster families and children living in institutional foster care.

Analyses on quantitative data will be conducted using the statistical software SPSS (IBM, Version 27.0).

All statistical analyses will consider the type of data available. Therefore, the primary outcome of the study will be assessed via the Friedman test as it is the non-parametric alternative to the one-way ANOVA with repeated measures, to compare the changes in reflective functioning scores between the pre-intervention, post-intervention, and one-year follow-up assessments, with the group (family foster care vs. residential programs) as a between-subjects factor and the time of assessment (pre, post, follow-up) as a within-subjects factor. In addition, the Mann–Whitney U test is used to compare differences between two independent groups (foster family and institutional foster care). To assess potential correlations regarding the child and caregivers’ capacity to mentalize, Spearman correlations (ρ) are conducted.

For the secondary outcome variables we choose a mixed-method approach for data analysis. Qualitative data from the “Patte-noir” will be processed via qualitative thematic analysis (Braun and Clarke, 2006). To provide an in-depth understanding of the psycho-social background of foster children and their foster caregiver, taking into account the caregivers’ social competences, their attitudes to drugs and drug addiction and the children’s mental state, quantitative methods, equal to the ones for the primary outcome variables, will be used.

This mixed-method approach was chosen to obtain different and multiple perspectives to validate the results and to build a comprehensive understanding.

## 3 Discussion

This study uses the concept of mentalization to identify affective and cognitive abilities of the caregiver-child dyad with the aim of compensating deficits on both sides with psychological-psychotherapeutic strategy. On the foster caregivers’ side, social structures and their anchoring in the social environment are addressed. Their prejudices are pointed out and questioned with the aim of providing suitable measures for change. Cassidy (2018) highlights that a foster caregiver of a traumatized child should be able to consistently empathize with the child, understanding that the child’s challenging behaviors may be a result from the child’s past experiences of abuse and neglect. As a further consequence, the child may be able to develop more secure and less disorganized attachment representations. On the children’s side, the emotional and affective background is illuminated, which at the same time reveals the risks for their future emotional, cognitive and social development. According to Suchman et al. (2020), cited in Paris et al. (2023), to the implementation of mentalization based approaches in research and practice of SUD patients treatment is still missing.

Initial findings from our research indicate that certain institutional foster caregivers exhibit low levels of mentalization capacity. Consequently, in collaboration with the Institute for Drug Prevention of the Office of Addiction and Drug Policy of Vienna, we will start in the near future the development for a mentalization-focused training program for institutional foster caregivers. The primary aims will be the enhancement of their reflective functioning, defined as the ability to mentalize, and to heighten sensitivity in interpersonal communication with children experiencing complex PTSD. The comprehensive training regimen will span several days of training sessions, supplemented by at least one mandatory reflection meeting within the initial year. The evaluation of the program’s effectiveness will rely on pre- and post-training measures of participants’ reflective functioning, utilizing an adapted version of the Reflective Functioning Questionnaire (RFQ-8). The detailed methodology and further findings will be disseminated in a forthcoming publication. The continued availability of this training program is an example for the successful transfer of results from a scientific study into practice to actually provide support to the sensitive target group.

The aim of publishing this study protocol is to inform the scientific community about the ongoing research and its transfer to clinical practice, and further to reach out to the community for exchanging ideas and to increase the coordination of research efforts.

However, the authors are very aware of some limitations of the study to mention. The small sample size will not allow do draw any gentrifications out of the results. Taken into account that the study is no RCT, historical control data will be used for comparison (Bizzi et al., 2022). One of the critical aspects of RCT is that “Intervention fidelity refers to the reliability and validity of the clinical interventions that are used in the randomized trial” (Cook and Thigpen, 2019, p. 64). Even though each group intervention of this study follows a strong ritualized procedure, its intervention fidelity cannot be comparable with an RCT on dosage of medication or manual based interventions. Still, the amount of the studies’ findings, due to the mixed-method design that is used, helps to deduce research questions for further studies. The study is carried out in a naturalistic setting. This can be considered as a strength as it increases its ecological validity.

As we are aware of the need for long-term support for this particularly sensitive group of subjects, all children have the option to attend the group intervention program more than once. Due to the third testing point after 12 months, the children who participate at the study will have to take a break until they attended the third measure time point before they can continue to participate at the group intervention.

## Data availability statement

The raw data supporting the conclusions of this article will be made available by the authors, without undue reservation.

## Ethics statement

The studies involving humans were approved by the Ethical Committee of the University of Vienna (#00295). The studies were conducted in accordance with the local legislation and institutional requirements. Written informed consent for participation in this study was provided by the participants’ legal guardians/next of kin.

## Author contributions

NS: Conceptualization, Data curation, Investigation, Methodology, Project administration, Resources, Writing – original draft. BL-S: Formal analysis, Supervision, Validation, Writing – review & editing.

## Footnotes

^1^www.dialog-on.at

^2^https://trialsearch.who.int/Trial2.aspx?TrialID=DRKS00027868
